# Factors influencing intravenous methylprednisolone pulse therapy in Chinese patients with isolated optic neuritis associated with AQP4 antibody-seropositive neuromyelitis optica

**DOI:** 10.1038/s41598-021-01109-5

**Published:** 2021-11-15

**Authors:** Sitong Guo, Hanqiu Jiang, Libin Jiang, Jingting Peng, Hongjuan Liu, Jiawei Wang, Wenbin Wei

**Affiliations:** 1 Beijing Tongren Eye Center, Beijing Tongren Hospital, Beijing Ophthalmology and Visual Sciences Key Laboratory, Capital Medical University, No 1, Dongjiaominxiang Street, Dongcheng District, Beijing, 100730 China; 2 Department of Neurology, Beijing Tongren Hospital, Capital Medical University, Beijing, 100730 China

**Keywords:** Optic nerve diseases, Vision disorders, Demyelinating diseases

## Abstract

This study investigated the factors influencing intravenous methylprednisolone pulse (IVMP) therapy for recovering visual acuity in Chinese patients with aquaporin-4 (AQP4) antibody-seropositive neuromyelitis optica-related optic neuritis (NMO-ON). This retrospective case series included 243 affected eyes of 182 patients (36 male, 146 female) diagnosed with NMO-ON in the Neuro-Ophthalmology Clinic of Beijing Tongren Hospital from September 2012 to September 2020. All patients with AQP4-antibody seropositivity had clinical manifestations of acute ON, excluding other diagnoses and received IVMP treatment at 500 mg/day or 1000 mg/day for 3 days. Primary outcome was the extent of improvement in logMAR visual acuity after IVMP treatment. The therapeutic influences of sex, age, baseline visual acuity, therapeutic intervals, and IVMP dose on acute NMO-ON were analysed. Chi-square tests, Mann–Whitney U-tests, Kruskal–Wallis tests, Spearman’s correlation coefficients, and multiple linear regression were used for statistical analysis. Age ranged between 7 and 80 years (median age, 44; interquartile range [IQR], 29–52) years. Among the 243 eyes, the median improvement in logMAR visual acuity was 0.3 (IQR, 0–0.9). Therapeutic efficacy of IVMP was significantly higher in female than in male patients (*Z* = 2.117, *P* = 0.034). The treatment effect gradually decreased with increase in age at onset (*Rs* = 0.157, *P* = 0.015), and visual improvement was significantly lower in patients aged > 50 years than in those ≤ 50 years (*Z* = 2.571, *P* = 0.010). When patients had low visual acuity at onset, improvements were more obvious (*rho* =  − 0.317, *P* < 0.001); however, final visual acuity was still low *(rho* = 0.688, *P* < 0.001). Therapeutic effect was negatively correlated with therapeutic intervals (*rho* = 0.228, *P* = 0.001). Dosage of methylprednisolone (1000 mg/day or 500 mg/day) did not significantly influence treatment efficacy (*Z* = 0.951 *P* = 0.342). Therefore, IVMP therapy can improve visual acuity in the affected eyes of patients with AQP4 antibody-seropositive NMO-ON with similar effect at 500 mg/day and 1000 mg/day doses. Sex, age at onset, and therapeutic intervals may influence the efficacy of IVMP in patients with NMO-ON.

## Introduction

Neuromyelitis optica (NMO) is an immune-mediated inflammatory disease of the central nervous system, typically manifesting with severe relapses of optic neuritis (ON) and longitudinally extensive myelitis. Aquaporin-4 (AQP4) antibody was the first validated diagnostic biomarker for NMO, with a diagnostic specificity of 68–91%^1^. As the most common inflammatory optic neuropathy in China^2^, NMO-related ON typically presents with severe visual loss, which mainly results from the impairment of the optic nerve in the acute stage and the cumulative effect of repeated relapses of the disease^3^.

Intravenous methylprednisolone pulse (IVMP) and plasma exchange are the main treatment methods in acute NMO-ON^4,5^, and some controversy remains surrounding the therapeutic effect of intravenous gamma globulin in acute NMO-ON^6,7^. Although some studies have shown that the therapeutic effect of IVMP combined with plasma exchange is better than that of IVMP alone in patients with NMO^8^, IVMP is the mainstay first-line treatment for acute NMO-ON in China due to the lack of plasma resources. Moreover, although IVMP is empirically used to treat acute attacks in patients with NMO, few large-scale studies have investigated the use of IVMP in isolated ON related to NMO. The influence of sex, age, and visual acuity at onset on the efficacy of IVMP treatment in patients with NMO-ON remains unknown. Therefore, in the present study, we aimed to examine the factors influencing the effectiveness of IVMP therapy on the recovery of visual acuity in patients with AQP4 antibody-seropositive NMO-ON. By contributing data from a large population of Chinese patients with NMO-ON, the results of our study may provide valuable insight into the treatment of NMO-ON using IVMP.

## Methods

### Patient enrolment

We designed a retrospective cohort study to evaluate the functional effectiveness of IVMP treatment in patients with NMO-ON. This study was conducted at the department of Neuro-ophthalmology in Beijing Tongren Hospital, Capital Medical University, China. Patient data were collected by reviewing medical records from September 2012 to September 2020. The study protocol was approved by the Ethics committee of Beijing Tongren Hospital affiliated with Capital Medical University (reference no: TRECKY2020-045) and adhered to the tenets of the Declaration of Helsinki. Informed consent was obtained from all participants or the legally authorised representatives of participants under 18 years of age.

Patients with a definitive diagnosis of NMO-ON with no evidence of symptoms or signs of lesions beyond the optic nerve were included in this study. The diagnostic criteria for NMO-ON aligned with the 2015 International Panel for NMO diagnosis^9^. The detailed inclusion criteria were as follows: (1) visual decrease, unilateral or bilateral, within 12 months; (2) a relative afferent pupillary defect (if the lesion was asymmetric); (3) ancillary laboratory tests with no evidence of infectious, hereditary, ischaemic, autoimmune, toxic, or metabolic aetiology; (4) brain/orbit MRI to assess the lesion in the optic nerve and rule out compression or intracranial lesions; (5) seropositivity for anti-AQP4 antibodies as measured using a cell-based assay, as previously reported^1^, employing IgG1-specific secondary antibodies; a dilution of 1:3200 was considered the maximum positive value and 1:10 as the cut off for positive and negative cases; (6) first episode of NMO-ON; (7) and no evidence of lesions beyond the optic nerve. We excluded patients who had severe heart, liver, and kidney diseases or other contraindications to glucocorticoid treatment.

A total of 182 patients with 243 affected eyes were identified in the present study. All patients were treated with intravenous methylprednisolone for 3 consecutive days, at a dose of 500 or 1000 mg/day, followed by a taper of oral prednisolone.

### Data collection

Demographic information such as sex, age at onset, baseline and final visual acuity, therapeutic interval from ON onset to initiation of IVMP therapy, and dose of methylprednisolone was collected using a standardised form. The primary outcome was the extent of visual improvement. Baseline and final visual acuity were defined as visual acuity before and 1–2 weeks after IVMP treatment, respectively. The decimal best-corrected visual acuity (BCVA) was converted to the logarithm of the minimal angle of resolution (logMAR) for statistical analyses using the following formula: logMAR =  − log (decimal acuity). Traditionally, patients undergoing visual acuity testing are semi-quantitatively classified into the categories of no light perception (NLP), light perception (LP), hand motion (HM), and finger count (FC). In reference to previous studies, the NLP, LP, HM, and FC categories were replaced by VA_logMAR_ = 2.9, 2.6, 2.3, and 2.0, respectively^9^.

### Statistical analysis

Statistical analyses were performed using IBM SPSS V.23.0 (Armonk, NY, USA). Comparisons between groups were performed using the Chi-square tests, Mann–Whitney U-test, Kruskal–Wallis tests, Spearman's correlation coefficients, and multiple linear regression. P-values < 0.05 were considered statistically significant.

## Results

### Demographic characteristics

The study included 182 patients with 243 eyes affected by NMO-ON. The age at onset ranged from 7 to 80 years (median, 44; interquartile range [IQR], 29–52 years). The ratio of male to female patients was 1:4.1. Ninety-six patients exhibited bilateral disease, simultaneously or successively, while the other patients exhibited unilateral disease until treatment. Among those with bilateral disease, 13 patients had simultaneous onset in both eyes, while 57, 14, nine, and three patients experienced the second onset at intervals of 1 year, 1–2 years, 3–5 years, and more than 5 years, respectively. Doses of methylprednisolone were 1000 mg/day in 171 affected eyes (70.4%) and 500 mg/day in 72 affected eyes (29.6%).

### Effects of IVMP treatment in patients with NMO-ON

**Figure 1:**
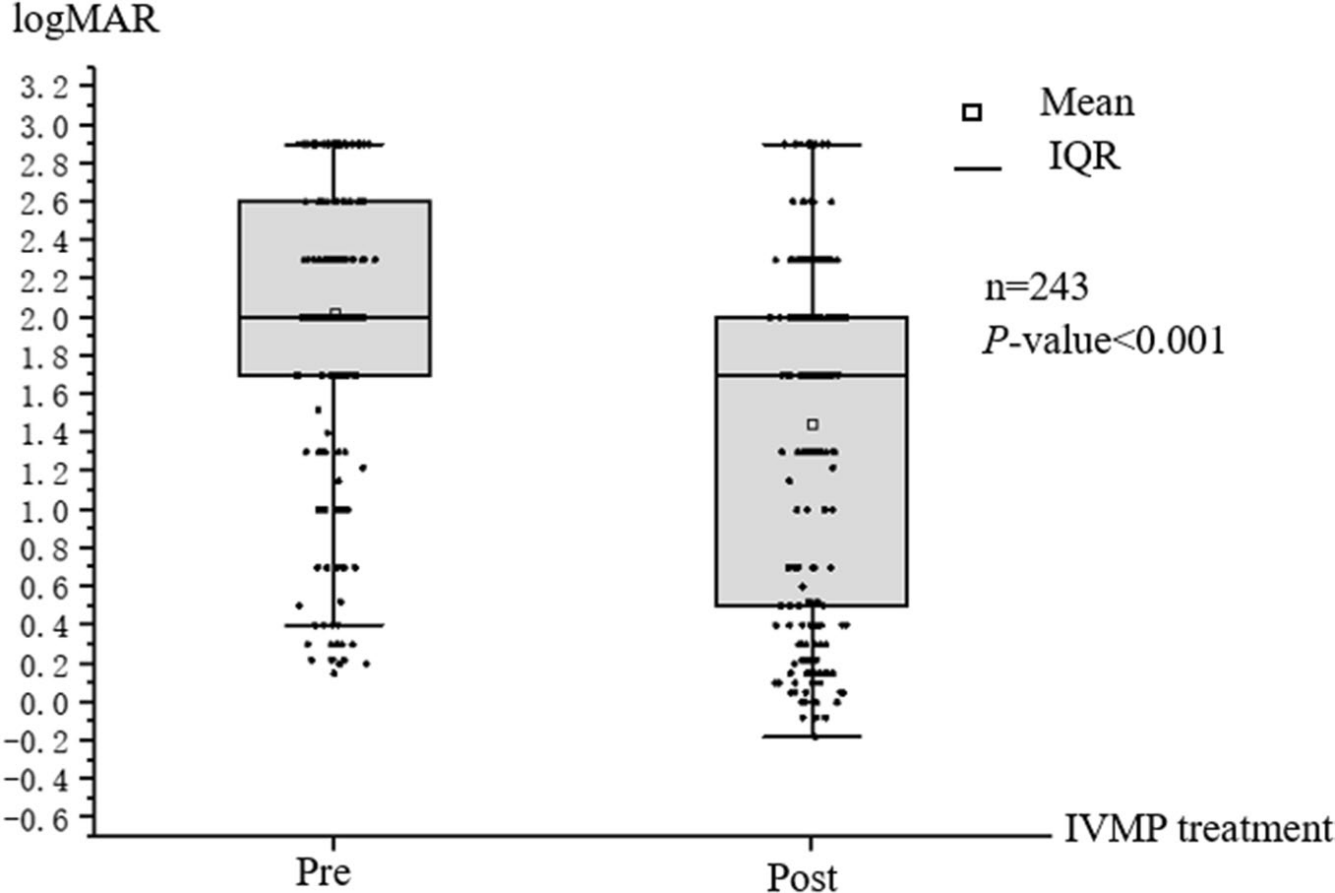
Distribution of logMAR visual acuity before and after IVMP treatment. Overall visual acuity was improved after IVMP treatment compared with before treatment.

After disease onset, the visual acuity of the patients decreased to different degrees: 57 eyes (23.5%) had no light perception, 18 eyes (7.4%) had light perception, 35 eyes (14.4%) responded to hand motion, and 68 eyes (28.0%) had finger counting abilities. The visual acuity of 196 eyes (80.7%) decreased to the state of blindness as defined by the World Health Organization (WHO)^10^ (logMAR visual acuity more than 1.3). Among them, 143 eyes (58.8%) remained blind after IVMP treatment. There was a significant difference in the distribution of vision before and after IVMP (Z = 10.808, *P* < 0.001) (Fig. 1).

Signs and symptoms improved or resolved in most patients after IVMP treatment. The median logMAR visual acuity at onset was 2.0 (IQR, 1.7–2.6), while that after treatment was 1.7 (IQR, 0.5–2.0). There was a significant difference in visual acuity before and after IVMP treatment (*Z* = 10.808, *P* < 0.001). After IVMP treatment, 29.6% of affected eyes exhibited no improvement, 27.2% exhibited an improvement less than 0.5, 24.7% exhibited an improvement more than 0.5, and 18.5% exhibited an improvement more than 1.0. The median improvement in logMAR visual acuity was 0.3 (IQR, 0–0.9).

### Factors influencing the efficacy of IVMP treatment

The median visual improvement in the affected eyes of female patients was 0.3 (IQR, 0–0.90), while that in male patients was 0.3 (IQR, 0–0.69). The therapeutic efficacy of IVMP was significantly better in female patients than in male patients (*Z* = 2.117, *P* = 0.034). The mean age at onset among female patients was 45 (IQR, 32.75–52.25) years, while that among male patients was 41 (IQR, 23.25–52.75) years. The mean primary visual acuity among female patients was 2.0 (IQR, 1.7–2.6), while that among male patients was 2.0 (IQR, 1.7–2.9). There was no significant difference in age or visual acuity at onset between male and female patients (*Z* = 1.161, *P* = 0.246) (*Z* = 0.615, *P* = 0.539).

**Figure 2:**
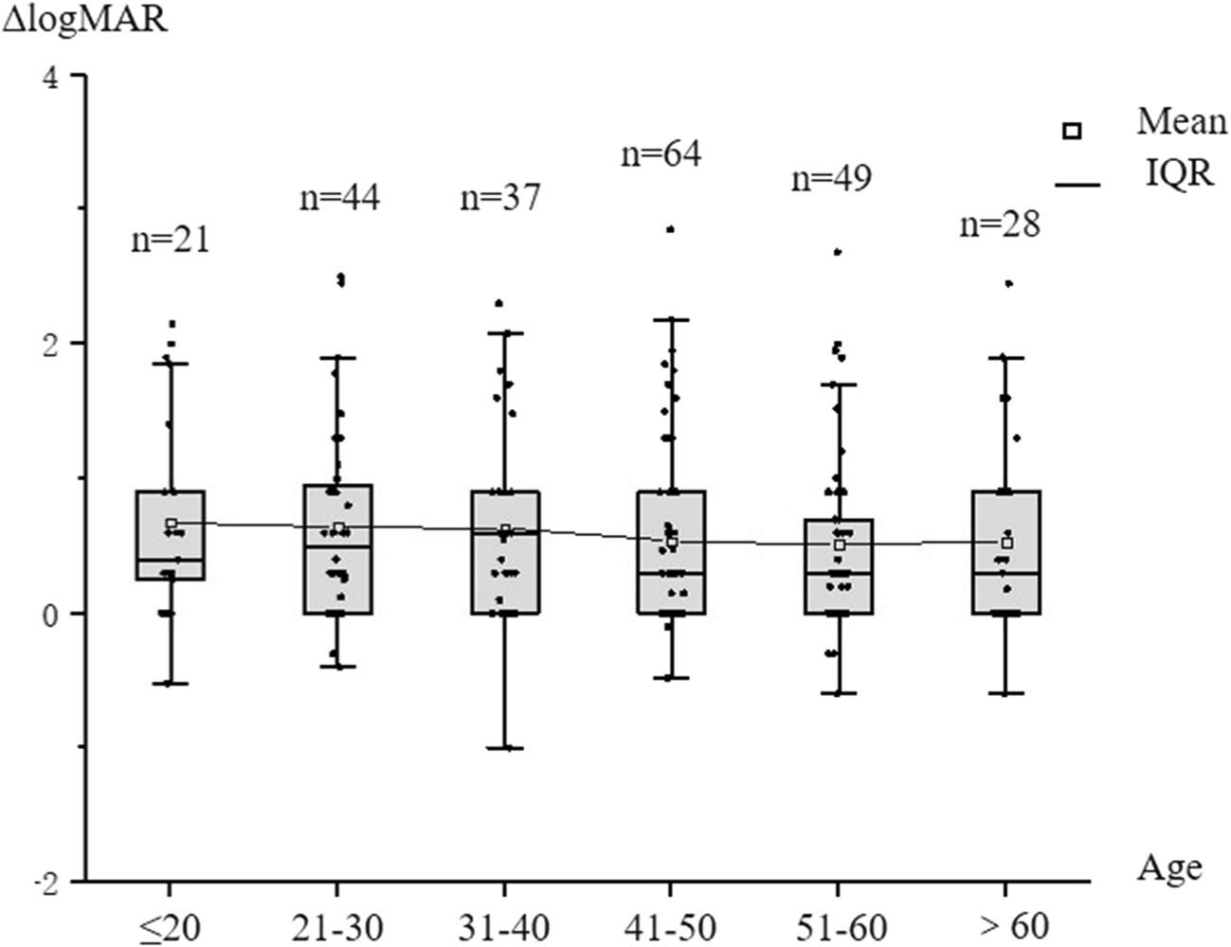
logMAR visual acuity improvement after IVMP treatment in different age groups. ΔlogMAR visual acuity decreased slightly with the increase of age.

The numbers of patients and affected eyes for specific age ranges were as follows: < 20 years old: 14 patients with 21 affected eyes; 21–30 years old, 32 patients with 44 eyes; 31–40 years old, 27 patients with 37 eyes; 41–50 years old, 51 patients with 64 eyes; 51–60 years old, 38 patients with 49 eyes; and > 60 years old, 20 patients with 28 eyes. The treatment effect gradually decreased as the age at onset increased (*rho* = 0.157, *P* = 0.015) (Fig. 2). Improvements in logMAR visual acuity IVMP treatment were significant in both those ≤ 50 years old (Z = 8.638 *P* < 0.001) and those aged > 50 years (Z = 6.604, *P* < 0.001). However, the therapeutic effect of IVMP was significantly lower in patients aged over 50 years than in patients aged ≤ 50 years (*Z* = 2.571, *P* = 0.010). The median improvement in logMAR visual acuity was 0.40 (IQR 0–0.90) in patients ≤ 50 years old and 0.30 (IQR 0–0.65) in patients > 50 years old. When the age cut-off was set to 40 years old, the median improvement in logMAR visual acuity was 0.44 (IQR 0–1.27) in patients ≤ 40 years old and 0.30 (IQR 0–0.90) in patients aged > 40 years. There was no significant difference in the treatment effect between the two groups (*Z* = 1.600, *P* = 0.110).

Visual acuity before treatment significantly influenced the therapeutic effect of IVMP treatment (*Z* = 13.515, *P* < 0.001). Correlation analysis revealed that low baseline visual acuity was associated with more obvious improvements in visual acuity after IVMP treatment (*rho* =  − 0.317, *P* < 0.001), and also with low final visual acuity *(rho* = 0.688, *P* < 0.001).

Therapeutic interval was defined as the days from ON onset to initiation of IVMP therapy, which ranged from 1 day to 12 months, with a median interval of 0.67 months. Treatment effects decreased as the interval increased (*rho* = 0.228, *P* = 0.001). Improvements in logMAR visual acuity were significant in those who received IVMP treatment within 0.67 months (*Z* = 8.085 *P* < 0.001) and beyond 0.67 months (*Z* = 7.178 *P* < 0.001).

### Effect of therapeutic dose on IVMP treatment

A total of 171 eyes received a methylprednisolone dose of 1000 mg/day, while 72 eyes received a dose of 500 mg/day, and there was no significant difference in therapeutic efficacy between the two groups (*Z* = 0.951 *P* = 0.342) (Table 1). In these two different methylprednisolone dose groups, there were no significant differences in sex (*X*^2^ = 0.170, *P* = 0.680), age (≤ 50 years and > 50 years, *X*^2^ = 0.925, *P* = 0.336), visual acuity in the affected eye at onset (*Z* = 0.251, *P* = 0.802), or therapeutic interval (*Z* = 0.362 *P* = 0.717). The total dose of methylprednisolone differed between the 500-mg and 1,000-mg groups (*Z* = 9.195, *P* < 0.001), but there was no correlation between the total dose of methylprednisolone and visual improvement (*rho* = 0.112 *P* = 0.082).

**Table 1:** Influence factors for therapeutic effect of IVMP therapy.

	Pre-logMAR VA Median (IQR)	Post-logMAR VA Median (IQR)	ΔlogMAR *P*-value
**Age (years)**			
≤ 50	2.0 (1.7–2.83)	1.7 (0.4–2.0)	0.010
> 50	2.3 (2.0–2.6)	2.0 (1.3–2.0)	
≤ 40	2.0 (1.7–2.83)	1.7 (0.43–2.0)	0.110
> 40	2.0 (1.7–2.6)	2.0 (0.7–2.0)	
**Sex**			
Male	2.0 (1.7–2.9)	2.0 (1.3–2.0)	0.034
Female	2.0 (1.7–2.6)	1.7 (0.4–2.0)	
**IVMP dose (mg/day)**			
500	2.0 (1.7–2.9)	2.0 (0.5–2.0)	0.342
1000	2.0 (1.7–2.9)	1.7 (0.52–2.0)	

Mann–Whitney U-test was performed in all comparisons.

*IVMP* intravenous methylprednisolone pulse, *IQR* interquartile range.

### Outcomes

The linear regression model indicated that sex, therapeutic intervals, age (cut-off age, 50 years), and baseline visual acuity influenced the therapeutic effect of IVMP treatment in patients with NMO-ON (Table 2).

**Table 2:** Linear regression analysis to therapeutic effect of IVMP therapy.

	B	SEB	Beta	t	*P*-value	95% CI	
						Lower bound	Upper bound
Age (cut-off of 50 years)	− 0.283	0.087	− 0.194	3.265	0.01	− 0.454	− 0.112
Sex (male)	0.304	0.99	0.181	− 3.059	0.002	0.108	0.500
Baseline VA	0.278	0.052	0.314	− 5.290	0.000	0.174	0.381
Therapeutic intervals	− 0.041	0.019	− 0.128	2.155	0.032	− 0.078	− 0.003
Dose	− 0.063	0.088	− 0.042	0.715	0.475	− 0.237	0.111

*IVMP* intravenous methylprednisolone pulse, *B* unstandardized regression coefficient, *SEB* standard error of the coefficient, *VA* visual acuity, *CI* confidence interval.

On analysing correlations based on another classification method (replacement of NLP, LP, HM, and FC categories with VA_logMAR_ = 3.0, 2.7, 2.3, and 1.85, respectively)^11^, we obtained the same results as above.

## Discussion

In this study, we retrospectively examined the clinical characteristics of 182 patients (243 eyes) with AQP4 antibody-positive NMO-ON. Our findings indicate that NMO-ON led to serious visual impairment, with visual acuity decreasing to finger counting, light perception, or even total lack of light perception in most patients. The age at onset was widely distributed from 7 to 80 years, in accordance with previously reported clinical features of NMO^12,13^. Binocular disease developed in 52.7% of patients, 72.9% of whom developed NMO-ON in the contralateral eye within 1 year of onset. Furthermore, 96.9% of patients had cumulative symptoms within 5 years.

As mentioned in the International Consensus Diagnostic Criteria for Neuromyelitis Option Spectrum Disorders (NMOSD) of 2015, serum AQP4-IgG is used as an important criterion for the diagnosis of NMOSD. Previous studies have also shown that patients with positive and negative AQP4-IgG have different clinical manifestations in terms of NMO severity, the therapeutic effect of glucocorticoids, and the tendency to relapse^2,14,15^. Expanded Disability Status Scale (EDSS) score was mainly used as the evaluation criteria for disease severity and improvement after treatment in NMO patients^15^. Many studies reported that in 45% of NMO patients with AQP4-IgG, the disease process starts with an acute attack of ON^15–17^ and patients with isolated ON may not reach an EDSS score higher than 4, even if complete bilateral visual loss is present. Therefore, it is necessary to evaluate the visual impairment of NMO patients presenting with isolated ON using professional ophthalmological/visual function evaluation criteria.

Overall, our findings indicated that median visual acuity after treatment was improved compared to that before treatment. After IVMP treatment, visual acuity improved by a median of 0.3 logMAR in patients with NMO-ON. However, the degree of improvement varied greatly. Some affected eyes did not respond to IVMP treatment, while some completely recovered from NLP to the baseline visual acuity before onset. Further, 72.8% of affected eyes had different responses to IVMP treatment, and more than 55.9% exhibited visual improvements of more than 0.5 logMAR after treatment. Our analysis revealed that the degree of visual improvement was associated with the patient’s age, sex, treatment intervals, and the degree of visual impairment at the time of onset.

Although patients with poor visual acuity before treatment exhibited greater improvements in visual acuity after treatment, their final visual acuity was poorer than those with better visual acuity before treatment. More than 80% of the affected eyes developed blindness before IVMP treatment and only 27% of them recovered visual ability to no less than 1.3 logMAR after IVMP treatment. This suggests that prompt and early IVMP treatment in patients with NMO-ON, before their visual acuity reached the nadir, may result in a better visual outcome. In contrast, eyes with low visual ability (including NLP, LP, HM, and FC) exhibited greater changes in logMAR values. For example, the change in logMAR value from NLP to LP is numerically equal to a change from 0.3 to 0.6 in standard visual acuity, but improvements in the latter may be more pronounced in the patient’s life. Given that results were the same when the two methods were compared, a more accurate and reliable method of evaluation is needed in clinical practice to objectively determine the degree of change in visual acuity in low-vision eyes^9,11^.

Prompt and early treatment is critical in IVMP therapy. A similar observation was made in several previous reports. Akaishi et al.^18^ found that early initiation of IVMP therapy earlier would affect the visual outcomes of AQP4-ON patients 1 year later. Another study showed that commencing IVMP treatment at ≤ 4 days of onset can increase the chance of full visual recovery, whereas start of treatment after more than 7 days was associated with a higher risk of poor visual recovery^19^. While improvements in visual acuity decreased as the interval between IVMP treatments increased, IVMP treatment produced improvements in visual acuity even after a 0.67-month therapeutic vacancy. These results remind us that IVMP treatment should be initiated as soon as possible in patients with NMO-ON. While earlier treatment will be associated with better improvements in visual acuity, high-dose glucocorticoid treatment should be considered even for patients presenting later in the disease course.

Although female sex is a strong risk factor of AQP4-IgG-positive NMO-ON^15^, the effectiveness of IVMP treatment were better in female patients than in male patients, and there were no significant differences in age at onset or baseline visual acuity between male and female patients. Another study demonstrated a similar result, with male patients likely to have visual outcomes less than 1.0 logMAR^2^. These findings suggest that male sex may adversely influence the response to IVMP therapy in patients with NMO-ON.

Increased age at onset is not only associated with a trend towards more motor disability and visual impairment^2,18,20,21^, but also contributed to low response to IVMP pulse therapy. Previous studies have reported that, in patients with NMO, decreases in EDSS scores are significant in those aged ≤ 40 years but not in those aged > 40 years, and the same results were obtained when the cut-off was set at 50 years of age^16^. Similarly, a Chinese study showed that age > 45 years at onset could suggest an inadequate response to high-dose methylprednisolone in NMOSD^17^. In the present study, although improvements in visual acuity were significant in all age groups (≤ 40 and > 40, ≤ 50 and > 50), the therapeutic effect was greater in patients ≤ 50 years old than in patients > 50 years old. This indicates that age is a crucial factor influencing IVMP response in patients with NMO, considering the poor efficacy of IVMP therapy and the adverse effects of high doses glucocorticoid in older adults with NMO-ON, alternative or superimposed treatment methods such as plasma exchange should be considered earlier.

NMO treatment has two main objectives: to control the inflammatory damage in acute attacks and to avoid relapses via maintenance treatment. The former is based on the use of high-dose intravenous corticosteroids. However, the effect of IVMP on NMO-ON remains to be determined due to a lack of large-scale multicentre studies. Previous studies have reported that the vast majority of patients with NMO exhibit treatment effects within 1 week after initiating IVMP therapy^16^. Therefore, in this study, we used the change in central visual acuity 1–2 weeks after IVMP treatment to evaluate treatment efficacy, and patients were followed up for more than 3 months to observe further increases or decreases in visual acuity. The standard of care for the treatment of acute ON or transverse myelitis associated with NMO is high-dose intravenous methylprednisolone at a daily dose of 1,000 mg for at least 3–5 days^4,5,22^. Due to differences in ethnicity and weight, whether large-scale glucocorticoid doses are suitable for Chinese patients with NMO-ON has rarely been investigated in large-sample studies in China. If a methylprednisolone dose of 500 mg/day can achieve the same therapeutic effect as a dose of 1000 mg/day, it would have clinical significance in that it would reduce the systemic side effects of corticosteroids. In this study, improvements in visual acuity following IVMP treatment did not significantly differ between patients treated with doses of 500 mg/day and 1000 mg/day, excluding the effects of sex, age, and interval. Although this result must be verified in large-scale prospective clinical studies, our data may provide reference values for future selection of glucocorticoid doses for Chinese patients with NMO-ON.

There were several limitations to the present study, including its retrospective, single-centre design. Second, we did not include various indicators of visual function such as assessments of the visual field, contrast sensitivity, and visual evoked potentials (VEP). Finally, as our study was not designed to investigate the long-term effects of IVMP, future studies should evaluate treatment efficacy over a longer follow-up period.

In summary, our findings demonstrate that IVMP treatment can effectively improve visual acuity in Chinese patients with AQP4 antibody-seropositive NMO-ON, to a limited degree. Moreover, the present results suggest that methylprednisolone doses of 500 mg/day and 1000 mg/day exert similar effects in patients with NMO-ON. In addition, female sex, younger age at onset, shorter therapeutic intervals, and worse baseline visual acuity are positively associated with improvements in visual acuity in patients with NMO-ON.

## Data Availability

All data analysed during this study are included in this published article.
